# Effects of Renin-Angiotensin-Aldosterone System Inhibition on Left Ventricular Hypertrophy, Diastolic Function, and Functional Status in Patients With Hypertrophic Cardiomyopathy: A Systematic Review

**DOI:** 10.7759/cureus.26642

**Published:** 2022-07-07

**Authors:** Hamza Akhtar, Hussein Al Sudani, Muhammad Hussein, Mehr un Nisa Farhan, Karim Elkholy

**Affiliations:** 1 Cardiology, Einstein Medical Center Philadelphia, Philadelphia, USA; 2 Internal Medicine, Einstein Medical Center Montgomery, East Norriton, USA; 3 Internal Medicine, St. Mary Medical Center, Trinity Health Mid-Atlantic, Philadelphia, USA; 4 Internal Medicine, Shaikh Khalifa Bin Zayed Al Nahyan Medical & Dental College, Lahore, PAK; 5 Internal Medicine, Einstein Medical Center Philadelphia, Philadelphia, USA

**Keywords:** systematic review, functional status, left ventricular diastolic dysfunction, left ventricular hypertrophy (lvh), raas inhibitors

## Abstract

The renin-angiotensin-aldosterone system (RAAS) plays a vital role in cardiovascular homeostasis by regulating blood pressure, salt, and water balance. The kidneys produce renin which converts angiotensinogen to angiotensin-1 (AT-I) and angiotensin-converting enzyme (ACE) to angiotensin-II (AT-II). AT-II binds to receptors in the adrenal cortex to release aldosterone. AT-II and aldosterone promote water and salt retention, vascular tone, and myocardial contractility. These physiological changes raise blood pressure and circulation. Reduced renal perfusion pressure sensed by baroreceptors and the sympathetic nervous system’s β-adrenergic receptors trigger renin release and RAAS activation. RAAS restores hemodynamic stability in pathological states associated with low perfusion. This adaptive response is important for restoring perfusion and hemodynamic stability, but prolonged RAAS activation has deleterious effects on the cardiovascular system. Long-term mineralocorticoid exposure has been linked to left ventricular hypertrophy (LVH) and remodeling. AT-II activates fibroblasts and cardiac myocytes to promote cardiac remodeling. Blocking RAAS can eliminate the long-term negative effects of RAAS activation. Direct renin inhibitors, ACE inhibitors, angiotensin receptor blockers, and aldosterone antagonists are RAAS blockers.

RAAS blockade improves mortality and hospitalization in systolic heart failure and acute myocardial infarction. RAAS blockade has not demonstrated the same benefits in other cardiac populations, such as those with preserved ejection fraction. Hypertrophic cardiomyopathy (HCM) causes LVH and asymmetric septal hypertrophy. When the outflow tract gradient exceeds 30 mmHg and is associated with septal hypertrophy, it is known as obstructive HCM. Dyspnea on exertion, syncope, and exertional angina are symptoms of HCM. RAAS activation worsens LVH by increasing blood pressure and by directly affecting cardiac myocytes with AT-II and aldosterone. RAAS blockade reverses myocardial fibrosis and slows HCM progression in animal models. We performed a meta-analysis of randomized clinical trials to further investigate the potential benefit of RAAS blockade in HCM patients. Although our findings included significant results for some of the RAAS blockade agents, these findings were not consistent throughout all the studies. Mavacamten, one of the newest treatments, has shown promising outcomes.

## Introduction and background

The renin-angiotensin-aldosterone system (RAAS) is a hormonal cascade that plays a vital role in cardiovascular homeostasis through the regulation of blood pressure, salt, and water balance. The pathway begins with the production of renin from the kidneys which leads to the conversion of angiotensinogen to angiotensin-I (AT-I) which is subsequently converted to the active peptide angiotensin-II (AT-II) by the enzyme angiotensin-converting enzyme (ACE). Among the targets of AT-II are the adrenal glands where it binds to receptors in the cortex and leads to the release of aldosterone. AT-II and aldosterone are the major end products of this pathway which promote water and salt retention and increase systemic vascular tone and myocardial contractility. The cumulative effect of these physiological changes is an increase in arterial blood pressure and effective circulating volume [1]. Major triggers for renin release and subsequent RAAS activation are decreased renal perfusion pressure which is sensed by renal baroreceptors and the sympathetic nervous system which exerts its effects through the β-adrenergic receptor [2]. Therefore, RAAS represents a compensatory physiological mechanism to restore hemodynamic stability in pathological states associated with low perfusion.

While this adaptive response is important in restoring perfusion and hemodynamic stability, it is now well recognized that prolonged activation of RAAS is associated with several deleterious effects on the cardiovascular system. Mineralocorticoid receptors are found outside the adrenal cortex such as on cardiac myocytes and long-term exposure has been associated with left ventricular hypertrophy (LVH) and remodeling [3]. Similarly, AT-II can promote cardiac remodeling by activating fibroblasts and cardiac myocytes [4]. Blockade of RAAS is an important therapeutic target that can eliminate the long-term negative effects associated with prolonged RAAS activation. RAAS blockers comprise a group of drugs that target the pathway at unique points and include direct renin inhibitors, ACE inhibitors, angiotensin receptor blockers, and aldosterone antagonists [5]. RAAS blockade in certain cardiac populations such as those with systolic heart failure and acute myocardial infarction has been associated with significant improvement in outcomes such as mortality and hospitalization [6,7]. However, the same beneficial effect of RAAS blockade has not been observed in other cardiac populations such as those with heart failure with preserved ejection fraction [8].

Hypertrophic cardiomyopathy (HCM) is common genetic cardiomyopathy characterized by LVH with or without asymmetric septal hypertrophy. An obstructive form is recognized when the outflow tract gradient exceeds 30 mmHg and is associated with pronounced septal hypertrophy. Symptoms of HCM overlap with those of heart failure and coronary artery disease and include dyspnea on exertion, syncope, and exertional angina. Activation of RAAS leads to a worsening of LVH both through increased blood pressure and through the direct effects of AT-II and aldosterone on cardiac myocytes. RAAS blockade in animal models has been shown to reverse myocardial fibrosis and potentially slow down the progression of HCM [9]. To further investigate the potential benefit of RAAS blockade in patients with HCM, we performed a meta-analysis of the randomized controlled trials (RCTs) that evaluated the effect of a RAAS blocker drug on outcomes in patients with HCM. We also included RCTs that examined the effect of mineralocorticoid receptor antagonists on outcomes in HCM patients, as this class of medications works by blocking the effect of aldosterone later in the pathway.

## Review

Methods

Search Strategy

A comprehensive literature search was conducted using the Preferred Reporting Items for Systematic Reviews and Meta-Analyses (PRISMA) to identify all relevant publications reporting the effects of RAAS blockade in patients with HCM. Candidate studies were identified by conducting a literature search of PubMed and Cochrane Central Register of Controlled Trials in the Cochrane Library databases from inception to May 2020 [10]. The actual search strategy was as follows: (((((((angiotensin converting enzyme inhibitors[MeSH Terms]) OR (aldosterone antagonist[MeSH Terms])) OR (mineralocorticoid receptor antagonist[MeSH Terms])) OR (direct renin inhibitors[MeSH Terms])) OR (angiotensin receptor neprilysin inhibitor[MeSH Terms])) AND (Hypertrophic cardiomyopahty[MeSH Terms])) OR (hypertrophic obstructive cardiomyopathy[MeSH Terms])) AND (randomized clinical trial[MeSH Terms]). Studies were reviewed individually and screened by titles and abstracts. Sub-studies or post-hoc analyses were excluded as they essentially examined the same trial population of the original trials included. Furthermore, reference lists from selected studies were manually screened to identify any additional relevant studies. The search was restricted to studies conducted among humans, and no language restriction was used.

Eligibility Criteria and Selection

Titles and abstracts were screened independently by two reviewers (H.A and K.L.B). Any discrepancies were resolved by consensus between the reviewing authors, or by consulting a third author (J.R) when an agreement could not be reached. The full text was reviewed when the articles did not provide enough information in the title and abstract. All screened articles were assessed for inclusion based on the following inclusion criteria: (1) study design was interventional; (2) presence of a control group; (3) study evaluated the effect of a RAAS blocker in patients with HCM; and (4) study reported relevant outcomes such as measures of LVH, diastolic function, and/or functional status.

Data Extraction

Data extraction was performed independently by two authors to compile information regarding patient and study characteristics as well as outcomes. Specific data items extracted for patient characteristics included age, sex, medications, and functional status. With regards to studying characteristics, extracted data items included authors, year, location, study design, intervention, sample size, and follow-up. To assess outcomes, we extracted data for LVH, diastolic function, and functional status. Data items extracted for LVH included left ventricular mass, left ventricular mass index, left ventricular fibrosis, N-terminal pro-peptide of collagen type I (PINP), and N-terminal pro-peptide of collagen type II (PIINP). Variables extracted to assess diastolic function included left atrial diameter, left atrial volume, N-terminal pro-brain natriuretic peptide (NT-proBNP), E/A, and E/E’. Finally, data for New York Heart Association (NYHA) class, peak oxygen consumption, bicycle ergometry, and six-minute walk test (6MWT) were extracted from included studies to assess functional status.

Results

**Figure 1:**
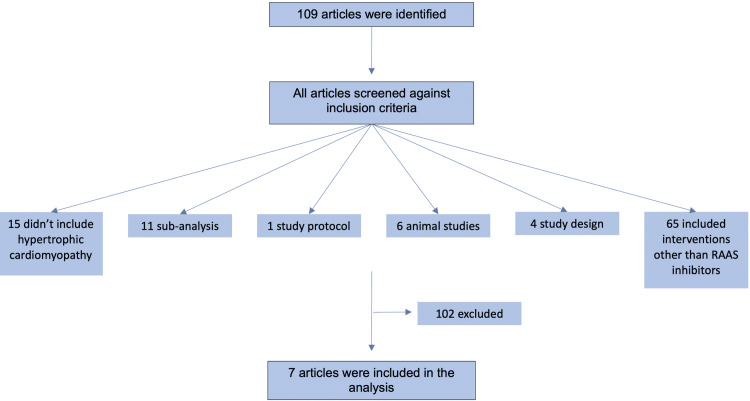
PRISMA diagram detailing the study identification and selection process. PRISMA: Preferred Reporting Items for Systematic Reviews and Meta-Analyses; RAAS: renin-angiotensin-aldosterone system

A total of 109 articles were identified utilizing our literature search; of these, 102 did not meet our inclusion criteria. The final number of included studies was seven (Figure 1).

Left Ventricular Hypertrophy

Three studies reported changes in left ventricular mass measured by cardiac magnetic resonance imaging (CMR) in patients receiving losartan [11-13]. Of these, Axelsson et al. [11] conducted the largest trial by randomizing 133 patients to receive either losartan or placebo. The authors did not find a significant reduction in left ventricular mass in the losartan group compared with the placebo (Table 1). Similarly, in the trials conducted by Shimada et al. [12] and Yamazaki et al. [13], there was no significant reduction in left ventricular mass noted in the intervention group compared with the placebo. However, Yamazaki et al. noted a significant reduction in the ratio of the final over initial left ventricular mass indicating a possible benefit on the natural course of hypertrophic non-obstructive cardiomyopathy (HNCM) (Table 1). Penicka et al. in their interventional study assessed changes in left ventricular mass by echocardiography in patients receiving candesartan over a period of 12 months [14]. They noted a significant decrease in left ventricular mass in the candesartan group compared with the placebo (Table 1). In another study, Maron et al. [15] did not observe a significant reduction in left ventricular mass as assessed by CMR after one year of spironolactone treatment compared with the placebo (Table 1).

**Table 1:** Left ventricular characteristics of selected studies. I: intervention group; C: control group; inter-group: intervention group against control group; LV: left ventricle; PINP: pro-collagen type I N-terminal pro-peptide; PIINP: pro-collagen type II N-terminal pro-peptide; PIIINP: pro-collagen type III N-terminal pro-peptide; PIP: pro-collagen type I

Study	Intervention	Sample size (n)	Measures of LV hypertrophy	Outcome	Statistical significance	
Shimada et al. [12]	Losartan	I: 11, C: 9	LV mass (g)	I: -5% (-11% to -0.9%)	I: Not reported	Inter-group: p = 0.06
				C: +5% (-4% to +21%)	C: Not reported	
			LV fibrosis (%)	I: -23% ± 45%	I: Not reported	Inter-group: p = 0.03
				C: +31% ± 26%	C: Not reported	
Yamazkazi et al. [13]	Losartan	I: 9, C: 10	LV mass (cm^3^)	I: 203 ± 47 to 190 ± 55	I: p = 0.07	Inter-group: p = 0.63
				C: 177±78 to 179 ± 45	C: p= 0.63	
Axelsson et al. [11]	Losartan	I: 58, C: 66	LV mass (g/cm^3^)	I: 103 ± 34 to 100 ± 42	I: p = 0.07	Inter-group: p = 0.60
				C:109 ± 33 to 105 ± 30	C: p= 0.0044	
			LV fibrosis (%)	I: +3% (0 to 10%)	I: p = 0002	Inter-group: p = 0.62
				C: +2% (0 to 6%)	C: p = 0.0004	
Maron et al. [15]	Spironolactone	I: 26, C: 27	LV mass index	I: -8.2 (111 ± 26 to 102 ± 21)	I: Not reported	Inter-group: p = 0.5
				C: -13[ 125±39 to 113±44]	C: Not reported	
			LV fibrosis (%)	I: +0.8% (1.1 ± 2.5 to 1.8 ± 2.9)	I: Not reported	Inter-group: p = 0.7
				C: +0.6% [2.5±3.3 to 2.8 ±4.1]	C: Not reported	
			PINP (µg/L)	I: -1.4 (2.1 ± 1.0 to 0.7 ± 1.2)	I: Not reported	Inter-group: p = 1.0
				C: -1.4 [ 2.1 ±1.2 to 0.6±1.3]	C: Not reported	
			PIINP (µg/L)	I: -2.8 (4.7 ± 2.0 to 2.0 ± 0.8)	I: Not reported	Inter-group: p = 0.8
				C: -3.0 (4.5 ± 2.5 to 1.6 ± 0.8)	C: Not reported	
Kawano et al. [16]	Valsartan	I: 11, C: 12	PIP (ng/mL)	I: 123 ± 63.1 to 102.8 ± 37.6	I: p < 0.05	Inter-group: Not reported
				C: 110.1 ± 40.5 to 121.6 ± 45.8	C: Not significant	
			PIIINP (ng/mL)	I: 0.63 ± 0.15 to 0.61 ± 0.07	I: Not significant	Inter-group: Not reported
				C:0.58±0.11 to 0.56±0.10	C: Not significant	
Penicka et al. [14]	Candesartan	I: 12, C: 11	LV mass (g)	I: 407 ± 139 to 344 ± 102	I: p < 0.001	Inter-group: p = 0.04
				C: 451 ± 228 to 449 ± 232	C: Not reported	

Left ventricular fibrosis as assessed by late gadolinium enhancement (LGE) with CMR was reported by a total of three studies [11,12,15]. Axelsson et al. noted an increase in myocardial fibrosis in both losartan and placebo groups; however, the increase did not differ significantly between the two groups (Table 1). Shimada et al. observed a statistically significant reduction in left ventricular fibrosis at one year in the losartan group compared with the placebo (Table 1). In the study by Maron et al., LGE by CMR did not yield significant differences in myocardial fibrosis after 12 months of treatment with spironolactone between the intervention and control groups (Table 1).

Two studies measured PINP and PIINP as markers of increased collagen synthesis and myocardial fibrosis [15,16]. Kawano et al. [16] observed a significant decrease in the levels of PIP, a marker of type I collagen synthesis in the intervention group after 12 months of treatment with valsartan. However, treatment with valsartan did not result in a significant change in serum levels of PIINP; a surrogate for type III collagen synthesis (Table 1). Maron et al. measured serum levels of PINP and PIINP in patients receiving treatment with spironolactone. The authors did not find a significant change in the levels of these markers of myocardial fibrosis after 12 months of treatment with spironolactone (Table 1).

Diastolic Function

Two of the three trials that evaluated the effect of losartan did not observe a significant improvement in measures of diastolic function. Furthermore, Axelsson et al. conducted a subgroup analysis of patients with an observed decrease in left ventricular mass and found no significant improvement in the parameters of diastolic function. The authors observed a significant increase in left atrial volume as assessed by CMR in study participants over 12 months (Table 2). On the other hand, in a study of 20 patients, Araujo et al. observed a significant reduction in left atrial diameter and left ventricular filling pressures as assessed by tissue Doppler-derived E/E’ ratio after six months of treatment with losartan compared with a placebo group [17] (Table 2). Penicka et al. studied the effect of candesartan in patients with HCM and observed significant improvement in left ventricular filling pressures as assessed by the E/E’ ratio in the treatment group but not in the placebo group (Table 2). Kawano et al. did not identify any significant improvement in measures of diastolic function such as left atrial diameter and the ratio of early to late diastolic filling peak flow velocities (E/A) in study participants after 12 months of treatment with valsartan (Table 2). Maron et al. evaluated the effect of treatment with 12 months of spironolactone on measures of diastolic function in patients with HCM. The authors did not find significant improvement in left atrial diameter or left ventricular filling pressures as assessed by the E/E’ ratio (Table 2).

**Table 2:** Diastolic function characteristics of selected studies. I: intervention group; C: control group; Inter-group: intervention group against control group; LA: left atrium; BNP: B-type natriuretic peptide; NT-proBNP: N-terminal pro-hormone B-type natriuretic peptide

Study	Intervention	Sample size (n)	Measures of diastolic function	Outcome	Statistical significance	
Shimada et al. [12]	Losartan	I: 11, C: 9	E/E’ (septal)	I: -7.5% (-13 to + 14)	I: Not reported	Inter-group: p = 0.79
				C: -8.2% (-22 to +11)	C: Not reported	
			E/E’ (lateral)	I: -6.7% (-19 to +19)	I: Not reported	Inter- group: p = 0.91
				C: +1.6% [-17 to +24]	C: Not reported	
			LA volume (mL)	I: - 9 ± 11	I: Not reported	Inter-group: p = 0.41
				C: +5 ± 12	C: Not reported	
			NT-proBNP	I: -7% (-38 to +8)	I: Not reported	Inter-group: p = 0.59
				C: -3% (-45 to +47)	C: Not reported	
Axelsson et al. [11]	Losartan	I: 58, C: 66	E/A	I: 0 ± 1.0	I: Not reported	Inter-group: p = 0.26
				C: -0.1 ± 0.5	C: Not reported	
			E/E’	I: -0.7 ± 5.2	I: Not reported	Inter- group: p = 0.28
				C: +0.2 ± 0.32	C: Not reported	
			LA volume index (mL/m^2^)	I: +5 ± 14	I: Not reported	Inter-group: p = 0.53
				C: +7 ± 14	C: Not reported	
			NT-proBNP	I: +4 (-1 to 14)	I: p = 0.037	Inter-group: p = 0.67
				C: +3 (-6 to 18)	C: p = 0.047	
Araujo et al. [17]	Losartan	I: 20 C: 10	E/A	I: 1.15 ± 0.4 to 1.38 ± 0.52	I: p = 0.03	Inter-group: Not reported
				C: 1.06 ± 0.5 to 1.09 ± 0.4	C: p = not significant	
			E/E’	I: 9.2 ±4.4 to 7.0 ± 2.8	I: p = 0.0002	Inter-group: Not reported
				C: 7.9 ± 2.6 to 8.3 ± 2.3	C: p = not significant	
			LA diameter (mm)	I: 44.6 ± 6.0 to 41.5 ± 7	I: p < 0.0001	Inter-group: Not reported
				C: Not reported	C: p = not significant	
			NT-proBNP (pg/mL)	I: 860 to 606	I: p = 0.001	Inter-group: Not reported
				C: 902 to 975	C: p = not significant	
Maron et al. [15]	Spironolactone	I: 26, C: 27	E/e’ (septal)	I: -0.6 (14 ± 4 to 13 ± 6)	I: Not reported	Inter-group: p = 1.0
				C: -2.0 (15 ± 7 to 13 ± 4)	C: Not reported	
			LA diameter (mm)	I: -0.3 (40 ± 6 to 40 ± 5)	I: Not reported	Inter-group: p= 0.8
				C: -0.2 (41 ± 5 to 40 ± 6)	C: Not reported	
Kawano et al. [16]	Valsartan	I: 11, C: 12	E/A	I: 0.99 ± 0.39 to 0.90 ± 0.42	I: Not reported	Inter-group: Not reported
				C: 0.83 ± 0.27 to 0.92 ± 0.33	C: Not reported	
			LA diameter	I: 41.1 ± 5.2 to 39.7 ± 4.4	I: p = not significant	Inter-group: Not reported
				C: 43.5 ± 6.7 to 43.3 ± 8.9	C: p = not significant	
			BNP (ng/mL)	I: 169 ± 231 to 165 ± 204	I: p = not significant	Inter-group: Not reported
				C: 156 ± 173 to 164 ± 154	C: p = not significant	
Penicka et al. [14]	Candesartan	I: 12, C: 11	E/E’	I: 13.5 ± 3.5 to 9.3 ± 1.3	I: p = not significant	Inter-group: p < 0.01
				C: 12.9 ± 3.9 to 12.3 ± 3.5	C: p = not significant	

Four studies reported data from NT-proBNP and one study measured BNP levels. Two studies on losartan did not observe changes in NT-proBNP levels in the treatment group compared with placebo. Araujo et al. observed a significant decrease in the levels of NT-proBNP in the losartan group which was not observed in the control group (Table 2). Kawana et al. did not find a significant decrease in BNP levels in the candesartan or the placebo group (Table 2).

Functional Capacity

Six studies reported changes in NYHA class as a measure of functional capacity. Two of the three trials that evaluated the effects of losartan did not find any significant improvement in the NYHA functional class after 12 months of treatment. Araujo et al. observed an improvement in asymptomatic status in 36% of patients with a symptomatic NYHA class in the losartan group. No improvement in NYHA class was noted in the placebo group (Table 3). Maron et al. found no significant improvement in NYHA class after 12 months of treatment with spironolactone (Table 3). Penicka et al. observed a non-significant decrease in NYHA class in six patients in the candesartan group compared with one patient in the placebo group (Table 3). 

**Table 3:** Functional capacity characteristics of selected studies I: intervention group; C: control group; Inter-group: intervention group against control group; NYHA class: New York Heart Association functional classification; 6MWD: six-minute walk test; METS: metabolic equivalent of task test; VO_2_: maximum rate of oxygen consumption measured during incremental exercise

Study	Intervention	Sample size (n)	Measures of functional capacity	Finding	Statistical significance	
Shimada et al. [12]	Losartan	I: 11, C: 9	NYHA class	Unchanged in all study participants	Not reported	
			6MWD (m)	I: +79 ± 416	I: Not reported	Inter-group: p = 0.76
				C: +32 ± 182	C: Not reported	
Axelsson et al. [11]	Losartan	I: 58, C: 66	NYHA class	No significant change between the two groups	Inter-group: p = 0.61	
			Mets (mL/kg/minute)	I: -0.2 ± 1.6	I: Not reported	Inter-group: p = 0.28
				C: +0.2 ± 1.7	C: Not reported	
Araujo et al. [17]	Losartan	I: 20, C: 10	NYHA class	I: 5/14 (36%) patients became asymptomatic	I: Not reported	Inter-group: Not reported
				C: 0/6 no patients became asymptomatic	C: Not reported	
Maron et al. [15]	Spironolactone	I: 26, C: 27	NYHA class	I: +0.1 (1.6 ± 0.7 to 1.7 ± 0.8)	I: Not reported	Inter-group: p = 0.8
				C: +0.1 (1.5 ± 0.6 to 1.6 ± 0.7)	C: Not reported	
			Peak VO_2_	I: 0 (30 ± 7 to 29 ± 8)	I: Not reported	Inter-group: p = 0.7
				C: +1.2 (28 ± 7 to 29 ± 6)	C: Not reported	
Penicka et al. [14]	Candesartan	I:12, C:11	NYHA class	I: 50% showed 1 point decrease	I: Not reported	Inter-group: p = 0.07
				C: 9% showed 1 point decrease	C: Not reported	
			Bicycle ergometry (seconds)	I: 574 ±151 to 751 ± 161	I: p < 0.001	Inter-group: p = 0.049
				C: 629 ± 149 to 603 ± 162	C: Not reported	

Other measures of functional capacity reported included the 6MWT, bicycle ergometer, and peak oxygen consumption with exercise. In the study by Axelsson et al., 130 of the 133 patients underwent exercise testing using a bicycle with a progressively increasing workload. The authors did not find a significant improvement in exercise capacity in the group receiving losartan compared with the placebo group (Table 3). Penicka et al. also assessed exercise tolerance using a bicycle ergometer and noted a significant increase in total exercise time in the candesartan group compared with the placebo group (Table 3). In the study by Maron et al. functional capacity was assessed by measuring peak oxygen consumption with exercise in the study participants. They did not find a significant improvement in peak oxygen consumption with exercise after 12 months of treatment with spironolactone compared with the placebo (Table 3). Shimada et al. observed no significant differences in the distance walked during a 6MWT in the losartan and placebo groups (Table 3). 

Discussion

Our study analysis demonstrates an improvement in left ventricular diastolic function, a decrease in NT-pro BNP level, and an improvement in patients’ symptoms into asymptomatic status after having NYHA class symptoms (observed by Araujo et al.) [17]. Candesartan demonstrated a significant decrease in the left ventricular mass, improved left ventricular diastolic function, and an increase in exercise tolerance total time (observed by Penicka et al.) [14]. Although valsartan showed a decrease in the synthesis of collagen I which was studied to be a contributor to cardiac fibrosis, it was not significant enough to improve LVH or patient symptoms (observed by Kawano et al.) [16]. A mineralocorticoid receptor blocker represented by spironolactone was observed to have no beneficial role in decreasing left ventricular mass, improving left ventricular diastolic function, and improving patient symptoms (observed by Maron et al.) [15].

In patients with the genetic predisposition to develop non-hypertrophic cardiomyopathy (NHCM) but no structural changes or symptoms, it has been suggested that the use of RAAS blockade may be more beneficial than when it is used in patients with left ventricular fibrosis and hypertrophy who have the genetic predisposition to HCM, which supports the hypothesis that ARBs can inhibit gene expression and reduce left ventricular mass in NHCMs [9,13,18]. In addition to their effects, different studies have shown that using ARBs and ACE inhibitors is safe [14,16,17].

We conclude through our study that HCM trials that investigated the effects of RAAS inhibitors on reducing LVH and fibrosis have demonstrated findings to be significant in some instances when using losartan or candesartan but not with valsartan; however, endpoints such as morbidity and mortality remain unknown. In addition, the mineralocorticoid receptor blocker represented by spironolactone did not show any evidence of playing a beneficial role in reducing left ventricular mass, improving left ventricular diastolic function, or relieving patient symptoms.

## Conclusions

According to the findings of our study, some of the RAAS blockade therapies have the potential to produce beneficial effects, such as the reduction of LVH, the improvement of left ventricular diastolic function, and the alleviation of symptoms in patients with HCM. However, the endpoints of morbidity and mortality are not yet known, and these beneficial effects were inconsistent throughout the studies.

## References

[1] Sparks MA, Crowley SD, Gurley SB, Mirotsou M, Coffman TM (2014). Classical renin-angiotensin system in kidney physiology. Compr Physiol.

[2] Skøtt O, Jensen BL (1993). Cellular and intrarenal control of renin secretion. Clin Sci (Lond).

[3] Catena C, Colussi G, Brosolo G, Novello M, Sechi LA (2015). Aldosterone and left ventricular remodeling. Horm Metab Res.

[4] Sadoshima J, Izumo S (1993). Molecular characterization of angiotensin II--induced hypertrophy of cardiac myocytes and hyperplasia of cardiac fibroblasts. Critical role of the AT1 receptor subtype. Circ Res.

[5] Rao MS (2010). Inhibition of the renin angiotensin aldosterone system: focus on aliskiren. J Assoc Physicians India.

[6] Xie W, Zheng F, Song X, Zhong B, Yan L (2016). Renin-angiotensin-aldosterone system blockers for heart failure with reduced ejection fraction or left ventricular dysfunction: network meta-analysis. Int J Cardiol.

[7] Al-Mallah MH, Tleyjeh IM, Abdel-Latif AA, Weaver WD (2006). Angiotensin-converting enzyme inhibitors in coronary artery disease and preserved left ventricular systolic function: a systematic review and meta-analysis of randomized controlled trials. J Am Coll Cardiol.

[8] Kuno T, Ueyama H, Fujisaki T, Briasouli A, Takagi H, Briasoulis A (2020). Meta-analysis evaluating the effects of renin-angiotensin-aldosterone system blockade on outcomes of heart failure with preserved ejection fraction. Am J Cardiol.

[9] Lim DS, Lutucuta S, Bachireddy P (2001). Angiotensin II blockade reverses myocardial fibrosis in a transgenic mouse model of human hypertrophic cardiomyopathy. Circulation.

[10] Higgins JP, Altman DG, Gøtzsche PC (2011). The Cochrane Collaboration's tool for assessing risk of bias in randomised trials. BMJ.

[11] Axelsson A, Iversen K, Vejlstrup N (2015). Efficacy and safety of the angiotensin II receptor blocker losartan for hypertrophic cardiomyopathy: the INHERIT randomised, double-blind, placebo-controlled trial. Lancet Diabetes Endocrinol.

[12] Shimada YJ, Passeri JJ, Baggish AL (2013). Effects of losartan on left ventricular hypertrophy and fibrosis in patients with nonobstructive hypertrophic cardiomyopathy. JACC Heart Fail.

[13] Yamazaki T, Suzuki J, Shimamoto R, Tsuji T, Ohmoto-Sekine Y, Ohtomo K, Nagai R (2007). A new therapeutic strategy for hypertrophic nonobstructive cardiomyopathy in humans. A randomized and prospective study with an angiotensin II receptor blocker. Int Heart J.

[14] Penicka M, Gregor P, Kerekes R, Marek D, Curila K, Krupicka J (2009). The effects of candesartan on left ventricular hypertrophy and function in nonobstructive hypertrophic cardiomyopathy: a pilot, randomized study. J Mol Diagn.

[15] Maron MS, Chan RH, Kapur NK (2018). Effect of spironolactone on myocardial fibrosis and other clinical variables in patients with hypertrophic cardiomyopathy. Am J Med.

[16] Kawano H, Toda G, Nakamizo R, Koide Y, Seto S, Yano K (2005). Valsartan decreases type I collagen synthesis in patients with hypertrophic cardiomyopathy. Circ J.

[17] Araujo AQ, Arteaga E, Ianni BM, Buck PC, Rabello R, Mady C (2005). Effect of Losartan on left ventricular diastolic function in patients with nonobstructive hypertrophic cardiomyopathy. Am J Cardiol.

[18] Masutomo K, Makino N, Fushiki MS (2001). Effects of losartan on the collagen degradative enzymes in hypertrophic and congestive types of cardiomyopathic hamsters. Mol Cell Biochem.

